# Efficacy, Safety, and Tolerability of Approved Combination BRAF and MEK Inhibitor Regimens for *BRAF*-Mutant Melanoma

**DOI:** 10.3390/cancers11111642

**Published:** 2019-10-24

**Authors:** Omid Hamid, C. Lance Cowey, Michelle Offner, Mark Faries, Richard D. Carvajal

**Affiliations:** 1 The Angeles Clinic and Research Institute, Los Angeles, CA 90025, USA; moffner@theangelesclinic.org (M.O.); mfaries@theangelesclinic.org (M.F.); 2 Baylor-Sammons Cancer Center, Texas Oncology, Dallas, TX 75246, USA; lance.cowey@usoncology.com; 3 Columbia University Irving Medical Center, New York, NY 10032, USA; rdc2150@cumc.columbia.edu

**Keywords:** binimetinib, BRAF inhibitor, *BRAF*-mutant melanoma, combination therapy, MEK inhibitor

## Abstract

No head-to-head studies exist comparing BRAF inhibitor/MEK inhibitor (BRAFi/MEKi) combination treatments for *BRAF-*mutant melanoma. A side-by-side analysis of randomized phase III trials is presented that evaluated dabrafenib/trametinib, vemurafenib/cobimetinib, and encorafenib/binimetinib. The baseline characteristics, efficacy, and safety were compared: COMBI-v (dabrafenib/trametinib versus vemurafenib); coBRIM (vemurafenib/cobimetinib versus vemurafenib); and COLUMBUS (encorafenib/binimetinib versus encorafenib and vemurafenib). Vemurafenib was the control arm in all studies. The data sources included literature databases, European public assessment reports, U.S. Food and Drug Administration review documents, and prescribing information. The baseline characteristics were similar, except for coBRIM, which had a higher proportion of patients with elevated lactate dehydrogenase (LDH) levels. The median progression-free survival (PFS) and overall response rate (ORR) were similar across the trials, although numerically higher values were observed with encorafenib/binimetinib. In contrast, the median overall survival (OS) was numerically longer with encorafenib/binimetinib (33.6 months) compared to dabrafenib/trametinib (25.6 months) and vemurafenib/cobimetinib (22.3 months). Among vemurafenib arms, PFS, ORR, and OS were similar, despite variations in the baseline LDH. Each combination displayed a unique safety profile, with higher incidences of pyrexia with dabrafenib/trametinib and photosensitivity reactions with vemurafenib/cobimetinib. This analysis of BRAFi/MEKi combinations for *BRAF*-mutant melanoma, while limited as not a direct head-to-head clinical trial, highlights the differences in tolerability and efficacy that may be useful for therapeutic decision making.

## 1. Introduction

Melanoma is the most lethal form of skin cancer [1,2], with a high incidence among young adults [1,2], and a prevalence that is increasing worldwide [3,4]. Approximately 50% of patients with metastatic melanoma have mutations in *BRAF*, and over 95% of these are in *BRAF* exon 15 at V600 [5].

The introduction of the BRAF inhibitors (BRAFi) vemurafenib in 2011 and dabrafenib in 2012 led to substantial improvements in progression-free survival (PFS) and overall survival (OS) compared with therapies available at that time [6,7]. However, the durability of the benefit observed with BRAFi is limited by acquired resistance to these agents via the reactivation of the mitogen-activated protein kinase (MAPK) pathway through transactivation of RAF homo- and heterodimers and subsequent MEK/ERK phosphorylation in cells with wild-type BRAF [8].

The dual inhibition of the MAPK pathway with the addition of a MEK inhibitor (MEKi) to BRAFi therapy was shown in subsequent clinical studies to further improve efficacy outcomes and reduce toxicities associated with MAPK pathway reactivation, including the incidence of secondary malignancies [9,10]. Three BRAFi/MEKi combinations (dabrafenib/trametinib, vemurafenib/ cobimetinib, and encorafenib/binimetinib) are considered the standard treatments for patients with advanced *BRAF*-mutant melanoma [11,12,13].

The experience with dabrafenib/trametinib and vemurafenib/cobimetinib combinations suggests that they have distinct safety profiles with adverse events (AEs) that limit tolerability, but comparable efficacy outcomes, with the median PFS of approximately 12 months and the median OS of approximately 24 months [9,10,14,15,16,17,18]. A third BRAFi/MEKi combination (encorafenib/ binimetinib) has recently become commercially available in several regions, including the United States [19,20] and the European Union [21,22], based on the results from the COLUMBUS study, a large randomized phase III trial in patients with advanced *BRAF*-mutant melanoma [23]. The COLUMBUS study reported a median PFS of 14.9 months with the encorafenib/binimetinib combination, with apparent differences in the rates of certain AEs associated with dabrafenib/trametinib and vemurafenib/cobimetinib [23].

Encorafenib has been shown to have a dissociation half-life of 30 h, compared with 2 h for dabrafenib and 0.5 h for vemurafenib [24]. Encorafenib also displayed stronger inhibition of proliferation compared with dabrafenib or vemurafenib in *BRAF* V600–mutant cell lines [24]. These pharmacological properties result in sustained target inhibition and higher potency, suggesting the potential for greater clinical efficacy [23,24]. Encorafenib also exhibits a larger window of anti-melanoma activity without paradoxical MAPK reactivation than dabrafenib and vemurafenib (paradox index; 50 versus 10 and 5.5, respectively) [25], which may correlate with the differences in some skin toxicities.

There have been no head-to-head comparisons of these combinations, and it is unlikely that any direct comparisons will be performed. In the absence of a definitive trial comparing these regimens and to inform therapeutic decisions, Daud et al. performed an indirect treatment comparison of the dabrafenib/trametinib and vemurafenib/cobimetinib regimens from randomized phase III trials of patients with *BRAF*-mutant melanoma [26]. Here, building on the previous analysis by Daud et al., this study presents a side-by-side comparison of the efficacy and safety of three BRAFi/MEKi combinations [9,10,23] and the common comparator vemurafenib in patients with *BRAF-*mutant melanoma.

## 2. Results

### 2.1. Baseline Characteristics

The data for the comparison of the baseline patient and disease characteristics were from the primary publications for each study, with data cutoff dates of 17 April 2014 for the COMBI-v trial, 9 May 2014 for the coBRIM trial, and 19 May 2016 for the COLUMBUS trial [9,10,23]. The patient and disease characteristics at baseline were generally well balanced between the treatment arms within each trial and were comparable across trials (Table 1), with the exception of the baseline serum lactate dehydrogenase (LDH) level [9,10,23]. The Eastern Cooperative Oncology Group (ECOG) performance scores were predominantly 0 (67–76%) or 1 (24–33%) and the metastatic status was predominantly M1c (59–65%) with 43% to 50% having three or more organs involved. LDH was elevated in a higher proportion of patients in the coBRIM trial (locally assessed; 46% vemurafenib/cobimetinib, 43% vemurafenib) than in the COMBI-v (centrally assessed; 34% dabrafenib/trametinib, 32% vemurafenib) or COLUMBUS trials (centrally assessed; 29% encorafenib/binimetinib, 27% vemurafenib) [9,10,23]. Although previous systemic immunotherapy was an exclusion criterion in the COMBI-v and coBRIM studies, in the COLUMBUS study there were eight (5%) patients in the encorafenib/binimetinib arm and seven (4%) in the vemurafenib arm who had received previous checkpoint inhibitors [9,10,23]. This small number of patients was not expected to have any impact on the comparison between the studies.

### 2.2. Efficacy

In the COMBI-v study, the data cutoff date for PFS, ORR, and DOR was 17 April 2014 [27], with the data cutoff date for OS being 13 March 2015 [28]. In the coBRIM study, the data cutoff date for PFS, ORR, and DOR was 16 January 2015 [29], with 28 August 2015 being the cutoff date for OS data [14]. In the COLUMBUS study, the data cutoff date was 19 May 2016 [23]. The cutoff date for OS data was 7 November 2017.

**Figure 1:**
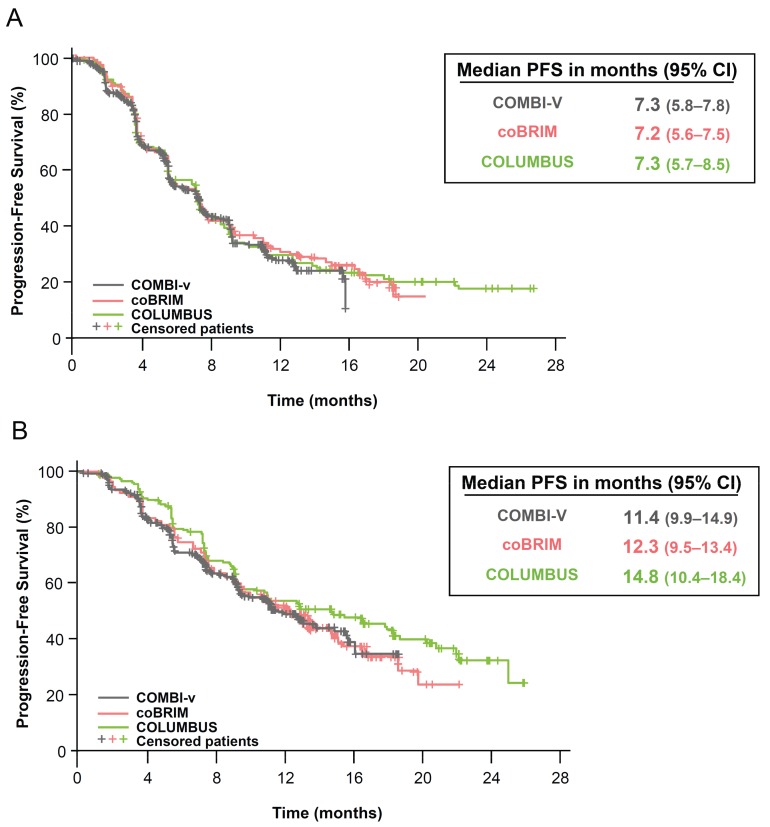
Local assessment of progression-free survival in (**A**) vemurafenib arms and (**B**) BRAFi/MEKi combination arms of COMBI-v, coBRIM, and COLUMBUS trials. CI indicates confidence interval; PFS, progression-free survival. Kaplan-Meier curves of progression-free survival from the vemurafenib arms and combination arms were superimposed.

The PFS, ORR, and DOR results for the treatment combinations in each trial were generally comparable, although numerically higher values for each parameter were observed in patients who received encorafenib/binimetinib compared with patients who received dabrafenib/trametinib or vemurafenib/cobimetinib (Table 2). The local assessment was compared for all three endpoints. The median DOR in the COLUMBUS vemurafenib arm (8.4 months) was consistent with the median values reported for vemurafenib in the other trials (7.5 months in COMBI-v and 9.2 months in coBRIM). Similarity was also observed in the median PFS (local review; 7.2–7.3 months; Figure 1A), ORR (49–51%), and OS (17.2–17.4 months) in the vemurafenib control arms across the trials [9,10,23]. In contrast, some numerical differences in the median PFS (11.4 [95% CI, 9.9, 14.9], 12.3 [9.5, 13.4], and 14.8 [10.4, 18.4] months were noted for the combination arms in COMBI-v, coBRIM, and COLUMBUS, respectively; Figure 1B) and ORR (64% [59%, 69%], 70% [64%, 75%], and 75% [68%, 81%], respectively) were observed among the combination treatment arms [9,10,23].

The median (95% CI) OS was 25.6 (22.6, not reached) months with dabrafenib/trametinib treatment in COMBI-v; 22.3 (20.3, not estimable) months with vemurafenib/cobimetinib treatment in coBRIM; and 33.6 (24.4, 39.2) months with encorafenib/binimetinib in COLUMBUS (Table 2) [14,28]. The hazard ratios (95% CI) were 0.66 (0.53, 0.81), 0.70 (0.55, 0.90), and 0.61 (0.47, 0.79), respectively.

The patients were followed for subsequent anticancer therapy after the study treatment discontinuation. The proportion of patients who received follow-up systemic therapy was lower in the COMBI-v study (72 patients [20%] in the dabrafenib/trametinib arm and 152 patients [43%] in the vemurafenib arm) relative to coBRIM (105 patients [57%] in the vemurafenib/cobimetinib arm and 125 patients [59%] in the vemurafenib arm) and COLUMBUS studies (80 patients [42%] in the encorafenib/binimetinib arm and 119 patients [62%] in the vemurafenib arm). In all three studies, ipilimumab was the most commonly administered follow-up anticancer therapy for patients in the combination and vemurafenib monotherapy arms [14,32,33] (see Table S1, which highlights the anticancer treatment by regimen following study drug discontinuation). The lack of immuno- oncology therapies in Europe at the time of the coBRIM and COMBI-v studies should be considered when interpreting these results.

### 2.3. Safety

The cutoff dates for the AE data presented here were 17 April 2014 for the COMBI-v trial, 19 September 2014 for the coBRIM trial, and 19 May 2016 for the COLUMBUS trial [23,31,33]. The AE summaries across the studies demonstrate that similar proportions of patients receiving the combination treatment regimens experienced serious AEs (34–37%), AEs leading to treatment discontinuation (13–15%), and dose interruptions/modifications (45–55%) (Table 3). The adverse events ≥grade 3 were reported at a higher incidence in patients receiving vemurafenib/cobimetinib (71%) compared with the other combination regimens (52% dabrafenib/trametinib, 58% encorafenib/binimetinib) (Table 3) [23,31,33].

The deaths due to events other than disease progression occurred in seven patients (4%) receiving encorafenib/binimetinib (two deaths due to unknown causes, and one death each due to cerebral hemorrhage, cerebral ischemia, completed suicide, euthanasia, and multiple organ dysfunction syndrome) [23]. Three patients (1%) receiving dabrafenib/trametinib experienced fatal AEs (two deaths due to cerebral hemorrhage and one due to brainstem hemorrhage) [23], and five patients (2%) receiving vemurafenib/cobimetinib experienced fatal AEs (one death each due to cardiac arrest, coma, pneumonia, *C. difficile* colitis, and one reported with a preferred term of death) [31,33].

**Figure 2:**
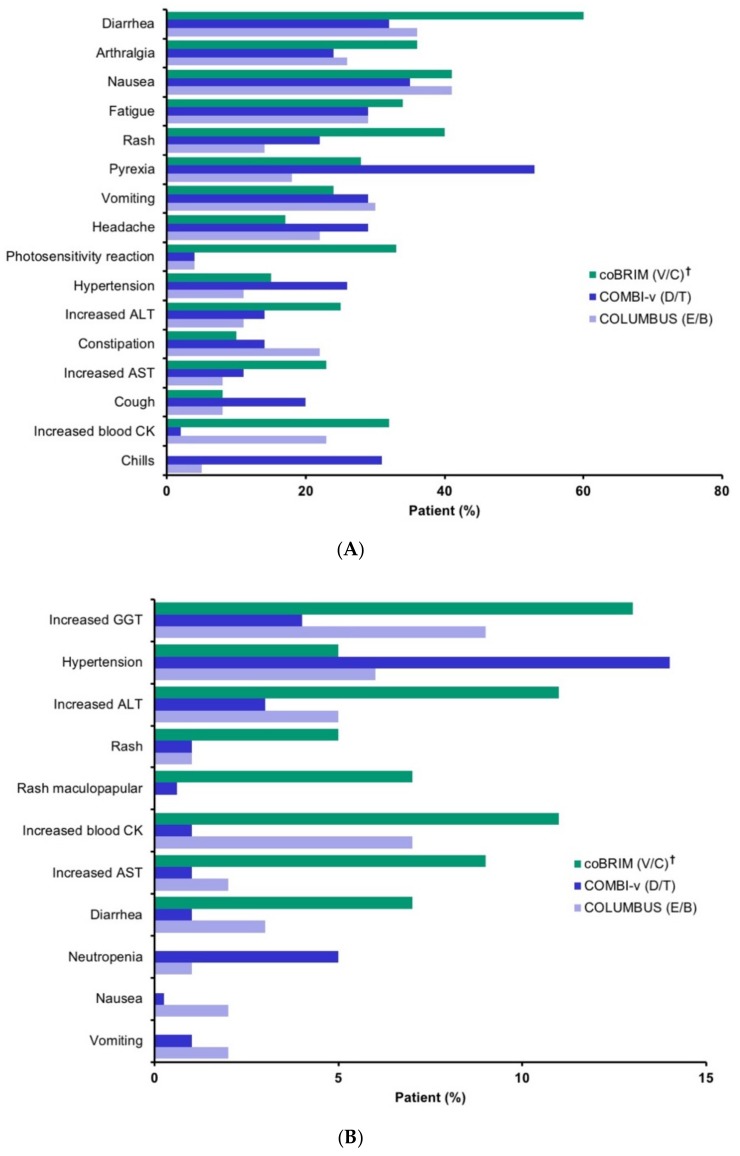
Adverse events (AEs) in (**A**) ≥20% of patients and in any combination treatment arm, and (**B**) grade 3/4 adverse events in ≥5% of patients in any combination treatment arm [23,31,33]. AE indicates adverse event; ALT, alanine transaminase; AST, aspartate transaminase; CK, creatinine phosphokinase; D/T, dabrafenib plus trametinib; E/B, encorafenib plus binimetinib; GGT, gamma-glutamyl transferase; NR, not reported; V, vemurafenib, V/C, vemurafenib plus cobimetinib. ^†^ The coBRIM data are from the safety update. Data for neutropenia, nausea, vomiting were not available. Events are presented in descending order of overall incidence in the combination arms.

The adverse events reported in ≥20% of patients in the BRAFi/MEKi combination arms in the three trials are summarized in Figure 2A. However, some laboratory tests were not conducted across the different studies and so cannot be appropriately contrasted. Additionally, there were significant differences in the way the AEs were monitored, further challenging the ability to compare rates. Despite this limitation, notable differences in the rates of the common AEs were identified, including relatively higher rates of diarrhea, arthralgia, rash, photosensitivity reaction, and increased alanine aminotransferase (ALT) with vemurafenib/cobimetinib; higher rates of pyrexia, hypertension, cough, and chills with dabrafenib/trametinib; and a higher rate elevated blood creatine phosphokinase with encorafenib/binimetinib (see Table S2, which presents AEs in ≥20% of patients in any combination treatment arm).

The grade 3/4 AEs reported in ≥5% of patients in the BRAFi/MEKi combination arms are summarized in Figure 2B. Most events were reported at a numerically higher incidence in patients receiving vemurafenib/cobimetinib compared with the other treatment combinations, with some events (increased ALT, increased aspartate aminotransferase, rash maculopapular) occurring at an incidence >5% higher in that group compared with the encorafenib/binimetinib and dabrafenib/trametinib groups (see Table S3, which illustrates grade 3/4 AEs reported in ≥5% of patients in any combination treatment arm).

The adverse drug reactions (ADRs) are defined by the International Conference on Harmonisation as “all noxious and unintended responses to a medicinal product related to any dose” and comprise events with at least a reasonable possibility of a causal relationship [35]. In reporting ADRs, individual AEs that are reported under different adverse event terms in the database but that represent the same phenomenon (eg. sedation, somnolence, drowsiness) are grouped together as a single adverse reaction term to avoid diluting or obscuring the true effect. Similarly, the AEs reported in more than one body system that appear to represent a common pathophysiologic event are grouped together to better characterize the reaction. For example, an allergic-type AE that has respiratory (wheezing) and dermatologic (rash, urticaria) manifestations would be classified as a single adverse reaction (eg. hypersensitivity) [36].

The adverse drug reactions for the dabrafenib/trametinib, vemurafenib/cobimetinib, and encorafenib/binimetinib treatment combinations are presented in Table 4. The relevant data on ADRs for the dabrafenib/trametinib combination were available only from the COMBI-d trial, a randomized double-blind phase III trial evaluating dabrafenib/trametinib versus placebo/trametinib in previously untreated patients with *BRAF*-mutant melanoma [16,18].

The ADRs from the COLUMBUS study include these preferred terms:

- Pyrexia includes pyrexia, increased body temperature, hyperpyrexia, hyperthermia;
- Peripheral edema includes peripheral edema, local swelling, localized edema, edema, peripheral swelling;
- Vomiting includes vomiting, retching;
- Arthralgia includes arthralgia, arthropathy, joint stiffness;
- Rash includes rash, exfoliative rash, erythematous rash, follicular rash, generalized rash, macular rash, maculo-papular rash, papular rash, pruritic rash, vesicular rash;
- Visual impairment includes visual impairment, blurred vision, reduced visual acuity;
- Serous retinopathy includes retinal detachment, chorioretinitis, chorioretinopathy, cystoid macular edema, macular retinal pigment epithelium detachment, retinal pigment epithelium detachment, macular detachment, macular edema, metamorphopsia, retinal disorder, retinal exudates, retinal edema, retinal pigment epitheliopathy, retinopathy, subretinal fluid;
- Hemorrhage includes rectal hemorrhage, hematochezia, hematuria, cerebral hemorrhage, epistaxis, hemorrhoidal hemorrhage, menorrhagia, metrorrhagia, retinal hemorrhage, conjunctival hemorrhage, gastric ulcer hemorrhage, gastrointestinal hemorrhage, hematospermia, hemorrhagic cyst, intracranial tumor hemorrhage, polymenorrhea, subdural hematoma, uterine hemorrhage, hemorrhagic diarrhea, hemoptysis, mucosal hemorrhage, occult blood, post procedural hemorrhage, postmenopausal hemorrhage, pulmonary alveolar hemorrhage, tumor hemorrhage, vaginal hemorrhage, wound hemorrhage.

The baseline characteristics for patients in COMBI-d were comparable to those of the patients who participated in the coBRIM and COLUMBUS trials [9,10,23]. The reported incidence of pyrexia was higher in the dabrafenib/trametinib group (57%) than in the other groups (28% and 18% in the vemurafenib/cobimetinib and encorafenib/binimetinib groups, respectively) [9,10,23]. Photosensitivity, rash (all grades and grade 3/4), diarrhea, and elevations in the liver function test parameters and blood creatine kinase (CK) occurred at a greater incidence in the vemurafenib/cobimetinib treatment group (≥20% higher incidence than the other combinations). The rates of ocular toxicity, including serous retinopathy, visual impairment, and uveitis, were similar across the COLUMBUS and coBRIM trials, but were not reported as routinely monitored in the COMBI-v trial.

## 3. Discussion

The introduction of BRAFi/MEKi combination regimens has proven to be a critical therapeutic breakthrough in the treatment of *BRAF*-mutant melanoma. Three such combinations are now currently available for use, and the results from a phase III trial in the most recently FDA-approved combination have been reported [23,27,29]. This side-by-side comparison has been performed to inform therapeutic decisions, particularly those relating to toxicities associated with each regimen.

The three phase III clinical trials included in this comparison have similar patient populations and study designs [9,10,23]. Importantly, each trial included a comparator arm of vemurafenib 960 mg twice daily. Most baseline characteristics were comparable across the trials. However, the proportion of patients with baseline LDH above the upper limit of normal in the coBRIM study (both treatment arms) was higher than that reported in the other trials. The percentages of patients exceeding the boundaries of normal provide an incomplete description of the values, particularly in the absence of details regarding the normal ranges used and the extent to which the values exceeded the normal range. Thus, it is difficult to determine the magnitude or to interpret the relevance of these differences. Nevertheless, LDH is a well-known prognostic factor in melanoma (as are factors such as M1c stage and ECOG performance status). The fact that vemurafenib performed almost identically across the trials may suggest that the prognostic characteristics of the patients across these trials were similar despite the differences observed in any single known prognostic factor. The similarity in the performance of vemurafenib extends beyond the median PFS and is reflected in the entire Kaplan–Meier curve for PFS across the trials where it was used as a comparator (Figure 1A). It would be difficult to account for this observation under the assumption that significant prognostic differences exist in the patient populations of these studies.

The potential clinical advantages observed with the encorafenib/binimetinib combination in this comparative analysis are, however, consistent with the pharmacokinetic and pharmacodynamic attributes of encorafenib when used in combination with binimetinib, as well as the clinical profile of encorafenib/binimetinib observed in the COLUMBUS trial, where encorafenib monotherapy demonstrated favorable clinical outcomes compared to vemurafenib [10,23,28]. In general, the survival rates for encorafenib/binimetinib were notably longer compared with other BRAFi/MEKi combination treatments. The PFS rates were similar among the three studies, with numerically higher values observed for encorafenib/binimetinib when compared to other combination treatments (PFS: encorafenib/binimetinib, 14.8 months; vemurafenib/cobimetinib, 12.3 months; dabrafenib/trametinib, 11.4 months). A similar trend was noted in the comparison of ORR (encorafenib/binimetinib, 75%; vemurafenib/cobimetinib, 70%; dabrafenib/trametinib, 64%) and median OS (encorafenib/ binimetinib, 33.6 months; vemurafenib/cobimetinib, 22.3 months; dabrafenib/trametinib, 25.6 months). With similar rates for vemurafenib across the three trials, this comparison provides potential insight into the relative clinical efficacy of various BRAFi/MEKi combination treatments. Of note, a comparative effectiveness analysis such as this is merely hypothesis generating. A myriad of explanations could also explain these findings, such as patient cohort variations, subsequent effective therapies (eg. PD-1 inhibitors, which were more widely available at the timing of the COLUMBUS trial compared to the earlier BRAF/MEK combination trials) and an improved clinical skill set of investigators in the use of these agents because BRAF and MEK inhibitors had been approved for several years prior to the timing of the COLUMBUS study.

The notable differences in the AE profile were seen among the BRAFi/MEKi combinations. Among these differences are clinically relevant and important toxicities that demonstrate a relatively higher incidence rate with dabrafenib/trametinib or vemurafenib/cobimetinib compared with encorafenib/binimetinib. Pyrexia associated with dabrafenib/trametinib is a major reason for discontinuation, dose interruption, and dose reduction [9,18], with patients often experiencing multiple episodes lasting a median of 3 days and sometimes requiring prophylactic treatment [18]. Photosensitivity reactions are common with vemurafenib/cobimetinib and require intensive management and patient education [10,14,29]. Both of these adverse events are less common with encorafenib/binimetinib [10,23,28].

Caution is necessary when interpreting data across clinical trials. Attempts to assess the relative risks and benefits of each combination regimen are complicated by several factors, especially the difference in reporting criteria for safety and adverse events based on publication and/or regulatory requirements. The COMBI-v trial was performed before the MEKi toxicity profile had been fully characterized and did not include routine monitoring for ocular toxicities and CK elevations, with the true incidence of these events unlikely to have been captured [9]. Additionally, the COMBI-v and coBRIM studies were conducted at a time when immunotherapies were largely unavailable. Furthermore, the differences in the study design and data collection challenge the ability for direct comparison. No attempt was made to adjust for any heterogeneity between the studies in terms of any patient characteristics or trial factors, and our comparisons are made through a simple side-by-side comparison. Although these differences exist, the consistency of the vemurafenib results as the control arm across efficacy endpoints including PFS, OS, and ORR in all three studies suggests that, although these factors are important, there is considerable homogeneity across the studies and overall clinical conclusions.

A clinical study directly comparing dabrafenib/trametinib, vemurafenib/cobimetinib, and encorafenib/binimetinib is unlikely to occur but would be necessary to confirm the signals of differentiated efficacy and safety identified in this across-trial comparison. There are no direct studies in the combinations and the closest is a direct comparison of BRAF monotherapy encorafenib versus vemurafenib. When used as a monotherapy, encorafenib showed improved clinical outcomes of ORR, OS, and PFS.

It is reasonable and useful to conduct this side-by-side comparison to help inform clinical practice, although the information discussed here does not show definitive evidence. It is intended to provide condensed information to help guide treatment decisions and to help provide context to clinicians when discussing treatment options with their patients. Further prospective, randomized, controlled studies comparing BRAF/MEK combinations would be needed to provide definitive guidance on the most efficacious and tolerable regimen.

## 4. Materials and Methods

A search was performed of regulatory and scientific literature to identify sources of clinical data to be used to compare the efficacy and safety of BRAFi/MEKi combination therapies for the treatment of *BRAF*-mutant metastatic melanoma. The full details of the literature search are outlined in Table S4. Four phase III studies were identified: coBRIM, a phase III trial of cobimetinib plus vemurafenib versus vemurafenib monotherapy in previously untreated patients with advanced *BRAF*-mutant melanoma [10]; COMBI-d, a phase III trial of dabrafenib and trametinib versus dabrafenib monotherapy in previously untreated patients with unresectable stage IIIC or stage IV melanoma with a *BRAF* V600E or V600K mutation [18]; COMBI-v, a phase III trial of dabrafenib plus trametinib versus vemurafenib monotherapy in previously untreated patients with unresectable stage IIIC or IV melanoma with *BRAF* mutation [9,14]; and COLUMBUS Part 1, a phase III trial of encorafenib plus binimetinib versus vemurafenib or encorafenib monotherapy in patients with *BRAF*-mutant melanoma [23]. Three of the identified studies (coBRIM, COMBI-v, and COLUMBUS Part 1), had vemurafenib 960 mg twice daily (BID) as a control arm [9,10,23], and were the chief sources of data for the comparisons presented here. Although the COMBI-d [18] was identified as part of the initial literature search, it was not included in the efficacy comparison of the cross-trial comparison due to the lack of a vemurafenib control arm. It should be noted, however, that the outcomes in COMBI-d were substantially similar to those in COMBI-v [9,16,18]. The key design features of the three included phase III trials are summarized in Table 5 [9,10,23]. To assist with the completeness of the cross-trial comparison, medical information requests were made to the registered sponsor of cobimetinib, vemurafenib, dabrafenib, trametinib, encorafenib, or binimetinib if any missing data were identified.

The data sources for the coBRIM and COMBI-v trials included primary and secondary publications of data, European public assessment reports (EPAR), U.S. Food and Drug Administration (FDA) medical review documents, and the current U.S. prescribing information (see Table S5, which illustrates the data sources and cutoff dates for COMBI-v, coBRIM, and COLUMBUS trials) [9,14,18,27,28,29,30,31,33,38]. The data sources for the COLUMBUS trial include a primary publication, U.S. prescribing information, and Array BioPharma data on file [23].

The efficacy outcomes presented are PFS, objective response rate (ORR), duration of response (DOR) per investigator assessment, and OS. The assessments of the tumor response across the studies were performed using Response Evaluation Criteria in Solid Tumors (RECIST) version 1.1 [10,23,28,39].

The safety comparisons were made using the adverse event (any untoward medical occurrence regardless of causal relationship) and adverse drug reaction (noxious and unintended responses with at least a reasonable possibility of a causal relationship) data [35].The adverse event data included the overall summaries (comprising all-grade, serious, and grade 3/4 AEs, and AEs leading to death, dose interruption/modification, and discontinuation) and the incidence of specific all-grade and grade 3/4 AEs by individual preferred terms per the MedDRA dictionary [40]. All studies utilized the National Cancer Institute (NCI) Common Terminology Criteria for Adverse Events (CTCAE) to assess severity. The coBRIM and COMBI-v trials used version 4.0, whereas the COLUMBUS trial used version 4.03. The primary source of data on adverse drug reactions was the available U.S. prescribing information [19,20,27,29,30,38]. The additional adverse event data for the dabrafenib plus trametinib combination was derived from the COMBI-d trial [27]. For the cobimetinib plus vemurafenib combination, the coBRIM trial was utilized [10]. The COLUMBUS study was utilized as a source of additional information for adverse events for the encorafenib plus binimetinib combination [23].

## 5. Conclusions

Our side-by-side comparison of efficacy and safety of dabrafenib/trametinib, vemurafenib/cobimetinib, and encorafenib/binimetinib in patients with *BRAF*-mutant melanoma identified important differences in efficacy, safety, and tolerability. This analysis of BRAFi/MEKi combinations for *BRAF*-mutant melanoma highlights the differences in tolerability and efficacy that may be useful for therapeutic decision making.

## Figures and Tables

**Table 1:** Baseline characteristics in the COMBI-v, coBRIM, and COLUMBUS trials [9,10,23].

Characteristics	COMBI-v		coBRIM		COLUMBUS	
	D/T	V	V/C	V	E/B	V
Intent-to-treat population	352	352	247	248	192	191
Age (year) Median (range)	55 (18–91)	54 (18–88)	56 (23–88)	55 (25–85)	57 (20–89)	56 (21–82)
Male sex, *n* (%)	208 (59)	180 (51)	146 (59)	140 (56)	115 (60)	111 (58)
ECOG performance score *n*/total *n* (%)						
0	248/350 (71)	248/352 (70)	184/243 (76)	164/244 (67)	136 (71)	140 (73)
1	102/350 (29)	104/352 (30)	58/243 (24)	80/244 (33)	56 (29)	51 (27)
2	0/350	0/352	1/243 (<1)	0/244	0	0
Metastatic status *n*/total *n* (%)						
M0	14/351 (4)	26/351 (7)	21 (9)	13 (5)	9 (5)	11 (6)
M1a	55/351 (16)	50/351 (14)	40 (16)	40 (16)	26 (14)	24 (13)
M1b	61/351 (17)	67/351 (19)	40 (16)	42 (17)	34 (18)	31 (16)
M1c	221/351 (63)	208/351 (59)	146 (59)	153 (62)	123 (64)	125 (65)
Number of organs involved *n*/total *n* (%)						
<3	177/351 (50)	201/352 (57)	NR	NR	105/192 (54)	104/191 (54)
≥3	174/351 (50)	151/352 (43)			87/192 (45)	87/191 (46)
Elevated LDH *n* /total *n* (%)	118/351 (34)	114/352 (32)	112/242 (46)	104/242 (43)	55/192 (29)	52/191 (27)
*BRAF* mutation *n*/total *n* (%)						
V600E	312/346 (90)	317/351 (90)	170/194 (88)	174/206 (84)	170/192 (89)	168/191 (88)
V600K	34/346 (10)	34 /351 (10)	24/194 (12)	32/206 (16)	22/192 (11)	23/191 (12)

D/T indicates dabrafenib plus trametinib; E/B, encorafenib plus binimetinib; ECOG, Eastern Cooperative Oncology Group; LDH, lactate dehydrogenase; NR, not reported; T, trametinib; V, vemurafenib; V/C, vemurafenib plus cobimetinib.

**Table 2:** Efficacy outcomes in the COMBI-v, coBRIM, and COLUMBUS trials.

Efficacy Outcome	COMBI-v [27,28]		coBRIM [30,31]		COLUMBUS [22,23,32]	
	D/T *n* = 352	V *n* = 352	V/C *n* = 247	V *n* = 248	E/B *n* = 192	V *n* = 191
PFS *^,†^, median (95% CI), mo	11.4 (9.9−14.9)	7.3 (5.8−7.8)	12.3 (9.5–13.4)	7.2 (5.6–7.5)	14.8 (10.4−18.4)	7.3 (5.7−8.5)
HR (95% CI)	0.56 (0.46−0.69)		0.58 (0.46−0.72)		0.49 (0.37−0.64)	
ORR * (95% CI), %	64 (59−69)	51 (46−56)	70 (64−75)	50 (44−56)	75 (68−81)	49 (42−57)
Median DOR * (95% CI), mo	13.8 (11.0−NR)	7.5 (7.3−9.3)	13.0 (11.1−16.6)	9.2 (7.5−12.8)	16.2 ** (11.1−20.4)	8.4 ** (5.8− 11.0)
Median OS (95% CI), mo	25.6 (18.3−NR)	17.2 (16.4−NR)	22.3 (20.3−NE)	17.4 (15.0−19.8)	33.6 (24.4−39.2)	16.9 (14.0−24.5)
HR (95% CI)	0.69 (0.53−0.89)		0.69 (0.54−0.88)		0.61 (0.47−0.79)	

CI indicates confidence interval; D/T, dabrafenib plus trametinib; DOR, duration of response; E/B, encorafenib plus binimetinib; HR, hazard ratio; NE, not estimable; NR, not reached; ORR, objective response rate; OS, overall survival; PFS, progression-free survival; V, vemurafenib; V/C, vemurafenib plus cobimetinib. * Per investigator assessment. ^†^ Updated PFS data (data cutoff: 13 March 2015) were subsequently reported for the COMBI-v trial (median PFS 12.6 months in the dabrafenib/trametinib arm and 7.3 months in the vemurafenib arm, HR 0.61, 95% CI 0.51, 0.73 [28]). ** Updated Investigator reported DOR (data cutoff: 7 November 2017).

**Table 3:** Overall summary of safety for the COMBI-v, coBRIM, and COLUMBUS trials.

AE type, *n* (%)	COMBI-v [9,27,28]		coBRIM [31,34]		COLUMBUS [22,23,32]	
	D/T *n* = 350	V *n* = 349	V/C *n* = 247	V *n* = 246	E/B *n* = 192	V *n* = 186
Any AE	343 (98)	345 (99)	244 (98.8)	240 (97.6)	189 (98)	185 (99)
Any serious AE	131 (37)	122 (35)	85 (34.4)	64 (26)	66 (34)	69 (37)
AE leading to death	3 (1)	3 (1)	5 (2)	3 (1.2)	6 (3)	2 (1)
Any grade ≥3 AE	183 (52)	221 (63)	176 (71.3)	146 (59.3)	111 (58)	118 (63)
Any dose interruptions/modifications	192 (55)	197 (56)	110 (44.5)	87 (35.4)	102 (53)	115 (62)
Discontinuation due to AE	44 (13)	41 (12)	37 (15)	20 (8.1)	29 (15)	32 (17)

AE indicates adverse event; D/T, dabrafenib plus trametinib; E/B, encorafenib plus binimetinib; V, vemurafenib; V/C, vemurafenib plus cobimetinib.

**Table 4:** Select adverse drug reactions, warnings, and precautions.

ADR, %	D/T * n = 209		V/C *n* = 247		E/B ^†^ *n* = 192	
	All Grades	Grade 3/4	All Grades	Grade 3/4	All Grades	Grade 3/4
General						
Pyrexia	57 ^‡^	7	28	2	18	4
Peripheral edema	25	1.4	12.6 [31]	NR	13	1
Chills	31	0	10	0	0	0
Gastrointestinal disorders						
Nausea	34	0.5	41	1	41	2
Vomiting	25	1.0	24	1	30	2
Diarrhea	30	1.4	60	6	36	3
Arthralgia	26	0.9	36 [31]	2.4 [31]	26	1
Skin						
Rash	42	0	73 [37]	17 [37]	22	1
Acneiform dermatitis	10 [33]	NR	16 [31]	2 [31]	4.4 [23]	0 [23]
PPE syndrome	5 [33]	NR	6 [34]	0 [34]	6.2 [23]	0 [23]
cuSCC ^§^	3	NR	6	NR	2.6	0
Basal cell carcinoma	3.3	NR	4.5	NR	1.6	0
LV dysfunction ^¶^	6 ^#^	NR	9 [34]	2 [34]	7	1.6 **
Creatine kinase increased ^††^	Not monitored ^‡‡^		79	14	58	5
Photosensitivity ^¶¶^	2 [31]	NR	46	4	4 [23]	0.4 [23]
Liver function tests ^††^						
ALT increased	44	3.8	68	11	29	6
AST increased	60	4.3	73	8	27	3
ALP increased	50	1.0	71	7	21	1
Hemorrhage	19	1.9	13	1	19	3.2
Ocular toxicity						
Serous retinopathy	Not monitored ^##^		26 ^†††^	NR	20 ^†††^	3
Visual impairment	NR	NR	15 ^‡‡‡^	<1	20 ^‡‡‡^	0
Uveitis	2 [33]	NR	2 [34]	NR	4	0
Venous thromboembolism	2.8 ^§§§^	NR	NR	NR	6	0
ECG QT prolonged	0.8 [33]	0 [33]	NR	1.6 [31]	0.5 ^###^	0
Hypertension	25	6	15	4	11	6

ADR indicates adverse drug reaction; ALP, alkaline phosphatase; ALT, alanine transaminase; AST, aspartate transaminase; cuSCC, cutaneous squamous cell carcinoma; ECG, electrocardiogram; KA, keratoacanthoma; LV, left ventricular; PPE, palmoplantar erythrodysaesthesia; NR, not reported. * ADRs in the prescribing information for D/T are based on data from the COMBI-d study [18]. ^†^ From the U.S. prescribing information for encorafenib unless otherwise noted, with the exception of PPE syndrome. ^‡^ Per the U.S. prescribing information: “Serious febrile reactions or fever of any severity complicated by severe rigors/chills, hypotension, dehydration, renal failure, or syncope, occurred in 17% (93/559) of patients with melanoma receiving TAFINLAR with trametinib. Fever was complicated by severe chills/rigors in 0.4% (2/559), dehydration in 1.8% (10/559), renal failure in 0.5% (3/559), and syncope in 0.7% (4/559) of patients.” ^§^ Including keratoacanthoma. ^¶^ LV dysfunction events were reported under the preferred term of left ventricular ejection fraction (LVEF) in the COMBI-d and coBRIM studies. ^#^ LVEF in COMBI-d study [18] was ≥10% decrease from baseline and <institutional LLN. ** All Grade 3. ^††^ Based on laboratory values. ^‡‡^ CK was not routinely monitored in COMBI-d study. ^¶¶^ Per the U.S. prescribing information: “Advise patients to avoid sun exposure, wear protective clothing and use a broad spectrum UVA/UVB sunscreen and lip balm (SPF ≥ 30) when outdoors”. For all ADRs of photosensitivity, the grouping includes solar dermatitis, sunburn, and photosensitivity reaction. ^##^ Per the U.S. prescribing information, routine monitoring of patients to detect asymptomatic retinal pigment epithelium detachment (RPED) was not conducted; therefore, the true incidence of this finding is unknown. ^†††^ Includes the preferred terms of chorioretinopathy, retinal detachment, detachment of retinal pigment, epithelium, macular edema, macular fibrosis, retinal disorder, retinopathy, subretinal fluid, and detachment of macular retinal pigment epithelium. ^‡‡‡^ Includes vision blurred, visual acuity reduced, visual impairment. ^§§§^ Deep vein thrombosis and pulmonary embolism. ^###^ In the COLUMBUS Trial, 1 of 192 patients (0.5%) who received encorafenib in combination with binimetinib reported a single incidence of an increase in QTcF to >500 ms.

**Table 5:** Overview of study designs for the COMBI-v, coBRIM, and COLUMBUS trials [9,10,23].

Study Design Characteristic	COMBI-v	coBRIM	COLUMBUS
Population	Unresectable locally advanced or metastatic melanoma with *BRAF* V600E/K mutation	Unresectable locally advanced or metastatic melanoma with *BRAF* V600 mutation	Unresectable locally advanced or metastatic melanoma with *BRAF* V600E and/or V600K mutation
Enrollment	704 patients (June 2012–Oct 2013)	495 patients (Jan 2013–Jan 2014)	577 patients (Dec 2013–April 2015)
Randomization	1:1	1:1	1:1:1
Treatments	dabrafenib 150 mg BID + trametinib 2 mg QD	vemurafenib 960 mg BID + cobimetinib 60 mg QD	encorafenib 450 mg QD + binimetinib 45 mg BID
	vemurafenib 960 mg BID	vemurafenib 960 mg BID	vemurafenib 960 mg BID encorafenib 300 mg QD *
Investigator/Patient blinding	no	yes	no
Prior systemic therapy permitted	none	none	first-line immunotherapy
Primary endpoint	OS	PFS (local)	PFS (central)
Secondary endpoints	PFS (local) ORR DOR	PFS (central) OS ORR DOR	PFS (local) OS ORR DOR TTR

BID indicates twice daily; DOR, duration of response; ORR, objective response rate; OS, overall survival; PFS, progression-free survival; QD, once daily; TTR, time to response. * Comparisons with encorafenib were secondary study endpoints and are not presented.

## Supplementary Materials

The following are available online at https://www.mdpi.com/2072-6694/11/11/1642/s1, Table S1: Anticancer Treatment by Regimen Following Study Drug Discontinuation, Table S2: Adverse Events in ≥20% of Patients in Any Combination Treatment Arm, Table S3: Grade 3/4 Adverse Events Reported in ≥5% of Patients in Any Combination Treatment Arm, Table S4: Scientific Literature Search Criteria, Table S5: Data Sources and Cutoff Dates for COMBI-v, coBRIM and COLUMBUS Trials.
