# Supplementary material for: Efficacy, Safety, and Tolerability of Approved Combination BRAF and MEK Inhibitor Regimens for BRAF-Mutant Melanoma
Source: Cancers (Basel). 2019 Oct 24;11(11):1642. doi: 10.3390/cancers11111642 (PMC6895913; doi:10.3390/cancers11111642)
Supplement: Supplementary file 1 [file cancers-11-01642-s001.pdf]

Supplementary Materials: Efficacy, Safety, and Tolerability of Approved Combination BRAF and MEK Inhibitor Regimens for *BRAF*-Mutant Melanoma

Omid Hamid, C. Lance Cowey, Michelle Offner, Mark Faries and Richard D. Carvajal

Table S1. Anticancer Treatment by Regimen Following Study Drug Discontinuation.

	**Dabrafenib + Trametinib** [1,2]		**Vemurafenib + Cobimetinib** [3]	**Encorafenib + Binimetinib** [4]
	**COMBI-v** † *n* **= 352**	**COMBI-d** *n* **= 209**	**coBRIM** *n* **= 183**	**COLUMBUS** *n* **= 192**
Any treatment	72 (20%)	101 (48%)	105 (57%)	80 (42%)
Immunotherapy	NR	117 (56%)	67 (37%)	NR
Any anti–PD-1/anti–PD-L1	NR	NR	28 (15%)	39 (20%)
Ipilimumab (anti-CTLA-4)	41 (12%)	86 (41%)	53 (29%)	33 (17%)
Nivolumab (anti-PD-1)	NR	15 (7%)	NR	NR
Pembrolizumab (anti–PD-L1)	4 (1%)	27 (13%)	NR	NR
anti-CTLA-4 + anti–PD-1/anti–PD-L1 ‡	NR	NR	4 (2%)	6 (3%)
Targeted therapies	NR	21 (10%) §	32 (18%)	NR
BRAFi + MEKi	NR	NR	15 (8%)	10 (5%) ¶
BRAFi	NR	NR	19 (10%)	11 (6%) #
MEKi	NR	NR	2 (1%)	NR
Chemotherapy	NR	37 (18%)	30 (16%)	14 (7%) **
Other	NR	NR	2 (1%) ††	5 (3%) ‡‡

NR = not reported. Data are *n* (%). PD-1 = programmed death cell receptor 1. PD-L1 = programmed death cell ligand 1. † Multiple uses of a type of therapy for an individual patient were only counted once in the frequency for that treatment category; patients mght have received multiple lines of treatment. Received by ≥2% of patients. ‡ Ipilimumab + nivolumab or ipilimumab + pembrolizumab. § Small-molecule targeted therapy. ¶ Dabrafenib + trametinib; dabrafenib + cobimetinib + trametinib; encorafenib + binimetinib; vemurafenib + cobimetinib; vemurafenib + protein kinase inhibitors. # Dabrafenib or vemurafenib. ** Abemaciclib; bleomycin; bleomycin + dacarbazine + lomustine + vincristine sulfate; carboplatin + dacarbazine + vinblastine sulfate; carboplatin + paclitaxel; carmustine + cisplatin + dacarbazine; carmustine + dacarbazine + hydroxycarbamide; cisplatin + dacarbazine; cisplatin + dacarbazine + vinblastine/vinblastine sulfate; cisplatin + temozolomide; cisplatin + vinblastine sulfate; dacarbazine; fotemustine; gemcitabine + treosulfan + trofosfamide; melphalan + norflox-tz; temozolomide; vinblastine sulfate. †† Other immunotherapy agents included granulocyte colony-stimulating factor and adoptive immunotherapy. ‡‡ Antineoplastic and immunomodulating agents; antineoplastic and immunomodulating agents + monoclonal antibodies; bevacizumab; bevacizumab + ipilimumab + nivolumab; binimetinib + buparlisib + encorafenib; binimetinib + capmatinib + encorafenib; binimetinib + encorafenib + ribociclib; dabrafenib + pembrolizumab; dabrafenib + pembrolizumab + trametinib; interferon; investigational drug; investigational drug + pembrolizumab; ipilimumab + vemurafenib; monoclonal antibodies; paclitaxel; radiotherapy; talimogene laherparepvec.

Table S2. Adverse Events in ≥20% of Patients in Any Combination Treatment Arm [5–7].

**Adverse Event, *n*, (%) ***	**COMBI-v**		**coBRIM**		**COLUMBUS**	
	**D/T**	**V**	**V/C**	**V**	**E/B**	**V**
	***n* = 350**	***n* = 349**	***n* = 247**	***n* = 246**	***n* = 192**	***n* = 186**
Diarrhea	112 (32)	131 (38)	148 (60)	76 (31)	70 (36)	63 (34)
Arthralgia	84 (24)	178 (51)	89 (36)	99 (40)	49 (26)	83 (45)
Nausea	121 (35)	125 (36)	102 (41)	62 (25)	79 (41)	63 (34)
Fatigue	101 (29)	115 (33)	85 (34)	80 (33)	55 (29)	57 (31)
Rash	76 (22)	149 (43)	98 (40)	94 (38)	27 (14)	54 (29)
Pyrexia	184 (53)	73 (21)	69 (28)	56 (23)	35 (18)	52 (28)
Vomiting	101 (29)	53 (15)	60 (24)	31 (13)	57 (30)	28 (15)
Headache	101 (29)	77 (22)	41 (17)	39 (16)	42 (22)	35 (19)
Photosensitivity reaction	13 (4)	78 (22)	82 (33)	45 (18)	8 (4)	45 (24)
Hypertension	92 (26)	84 (24)	37 (15)	19 (8)	21 (11)	21 (11)
Increased ALT	48 (14)	61 (17)	61 (25)	44 (18)	21 (11)	14 (8)
Constipation	48 (14)	61 (17)	24 (10)	26 (11)	42 (22)	12 (6)
Increased AST	40 (11)	45 (13)	58 (23)	31 (13)	16 (8)	4 (2)
Cough	69 (20)	34 (10)	19 (8)	30 (12)	16 (8)	13 (7)
Increased blood CK	7 (2)	1 (<1)	80 (32)	7 (3)	44 (23)	4 (2)
Chills	110 (31)	27 (8)	NR	NR	9 (5)	10 (5)

AE = adverse event; ALT = alanine transaminase; AST = aspartate transaminase; CK = creatinine phosphokinase; D/T = dabrafenib plus trametinib; E/B = encorafenib plus binimetinib; NR = not reported; V = vemurafenib, V/C = vemurafenib plus cobimetinib. * Events are presented in descending order of overall incidence in the combination arms.

Table S3. Grade 3/4 Adverse Events Reported in ≥5% of Patients in Any Combination Treatment Arm [5–7].

**Adverse event, % ***	**COMBI-v**		**coBRIM †**		**COLUMBUS**	
	**D/T**	**V**	**V/C**	**V**	**E/B**	**V**
	***n* = 350**	***n* = 349**	***n* = 247**	***n* = 246**	***n* = 192**	***n* = 186**
Increased GGT	4	5	13	10	9	3
Hypertension	14	9	5	2	6	3
Increased ALT	3	4	11	6	5	2
Rash	1	9	5	6	1	3
Rash maculopapular	<1	4	7	5	0	4
Increased blood CK	1	<1	11	0	7	0
Increased AST	1	3	9	2	2	2
Diarrhea	1	<1	7	<1	3	2
Neutropenia	5	1	–	–	1	1
Nausea	<1	<1	–	–	2	2
Vomiting	1	<1	–	–	2	1

AE = adverse event; ALT = alanine transaminase; AST = aspartate transaminase; CK = creatinine phosphokinase; D/T = dabrafenib plus trametinib; E/B = encorafenib plus binimetinib; GGT = gamma-glutamyl transferase; V = vemurafenib; V/C = vemurafenib plus cobimetinib.* Events are presented in descending order of overall incidence in the combination arms.† Data for neutropenia, nausea, vomiting were not available.

Table S4. Scientific Literature Search Criteria.

- Literature Databases
  - National Library of Medicine
  - EMBase
  - OVID
  - BIOSIS
- Search Terms
  - Clinical Trial
    - Clinical Trial; Clinical Trial, Phase II; Clinical Trial, Phase III; Clinical Trial, Phase IV; Comparative Study; Controlled Clinical Trial; Multicenter Study; Observational Study; Randomized Controlled Trial
- MeSH Headings
  - Melanomas
  - Malignant Melanoma
  - Malignant Melanomas
  - Melanoma, Malignant
  - Melanomas, Malignant
- Boolean Terms
  - BRAF-mutant Malignant Melanoma
  - BRAF-mutant
  - Combination Treatment
  - BRAF Inhibitor
  - MEK Inhibitor
  - BRAF / MEK Inhibitor
  - BRAF and MEK Inhibitor
- Search Type
  - Boolean-based AND / OR analysis
- Study Population(s)
  - All
- Age
  - All
- Study Ethnicity
  - All
- Species
  - Human
- Language(s)
  - English
- Publication Types
  - All
- Journal Categories
  - All
- Timeframe
  - January 1, 2000 to December 1, 2018
- Exclusions
  - Clinical Trial
    - Clinical Trial, Phase I; Pre-clinical
  - Publication Types
    - Literature Review; Case Studies; Secondary Analysis;
  - Search terms
    - None
  - Population
    - None

Table S5. Data Sources and Cutoff Dates for COMBI-v, coBRIM and COLUMBUS Trials.

	**COMBI-v**		**coBRIM**		**COLUMBUS**	
	**Source**	**Data Cutoff**	**Source**	**Data Cutoff**	**Source**	**Data Cutoff**
Baseline characteristics	Robert et al. primary publication [2]	17 Apr 2014	Larkin et al. primary publication [8]	9 May 2014	Dummer et al. primary publication [5]	19 May 2016
			Efficacy			
PFS, OS, ORR, DOR,	U.S. dabrafenib label (6/2017) [9] Robert et al. primary publication [2]	17 Apr 2014	U.S. cobimetinib label (5/2016) [10] EPAR [7]	16 Jan 2015 [7]	Dummer et al. primary publication [5]	19 May 2016
	Robert et al. 2015 [11] EPAR [6]	13 Mar 2015	Ascierto et al. 2016 [3]	28 Aug 2015	Dummer OS [4]	07 Nov 2017
			Safety			
Safety summary, most common AEs, Grade 3/4 AEs Most common AEs, Grade 3/4 AEs	Robert et al. primary publication [2]	17 Apr 2014	Larkin et al. primary publication [8]	19 Sept 2014 (update)	Dummer et al. primary publication [5]	19 May 2016
	dabrafenib/trametinib		vemurafenib/cobimetinib		encorafenib/binimetinib	
ADRs *	U.S. trametinib label (6/2017) [12]	12 Jan 2015	U.S. cobimetinib label (5/2016) [10]	19 Sep 2014 (update)	U.S. encorafenib label (6/2018) [13,14]	19 May 2016

ADR = adverse drug reaction; AE = adverse events; DOR = duration of response; EPAR = European public assessment report; NA = not applicable; ORR = objective response rate; OS = overall survival; LDH = lactate dehydrogenase; PFS = progression-free survival. * Main sources of data; other sources noted in Table 8.

Reference

1. Long, G.V.; Flaherty, K.T.; Stroyakovskiy, D.; Gogas, H.; Levchenko, E.; de Braud, F.; Larkin, J.; Garbe, C.; Jouary, T.; Hauschild, A., et al. Dabrafenib plus trametinib versus dabrafenib monotherapy in patients with metastatic BRAF V600E/K-mutant melanoma: long-term survival and safety analysis of a phase 3 study. *Ann. Oncol.* **2017**, *28*, 1631–1639, doi:10.1093/annonc/mdx176.
2. Robert, C.; Karaszewska, B.; Schachter, J.; Rutkowski, P.; Mackiewicz, A.; Stroiakovski, D.; Lichinitser, M.; Dummer, R.; Grange, F.; Mortier, L., et al. Improved overall survival in melanoma with combined dabrafenib and trametinib. *N. Engl. J. Med.* **2015**, *372*, 30-39, doi:10.1056/NEJMoa1412690.
3. Ascierto, P.A.; McArthur, G.A.; Dreno, B.; Atkinson, V.; Liszkay, G.; Di Giacomo, A.M.; Mandala, M.; Demidov, L.; Stroyakovskiy, D.; Thomas, L., et al. Cobimetinib combined with vemurafenib in advanced BRAF(V600)-mutant melanoma (coBRIM): updated efficacy results from a randomised, double-blind, phase 3 trial. *Lancet Oncol.* **2016**, *17*, 1248-1260, doi:10.1016/s1470-2045(16)30122-x.
4. Dummer, R.; Ascierto, P.A.; Gogas, H.J.; Arance, A.; Mandala, M.; Liszkay, G.; Garbe, C.; Schadendorf, D.; Krajsova, I.; Gutzmer, R., et al. Overall survival in patients with BRAF-mutant melanoma receiving encorafenib plus binimetinib versus vemurafenib or encorafenib (COLUMBUS): a multicentre, open-label, randomised, phase 3 trial. *Lancet Oncol.* **2018**, *19*, 1315-1327, doi:10.1016/s1470-2045(18)30497-2.
5. Dummer, R.; Ascierto, P.A.; Gogas, H.J.; Arance, A.; Mandala, M.; Liszkay, G.; Garbe, C.; Schadendorf, D.; Krajsova, I.; Gutzmer, R., et al. Encorafenib plus binimetinib versus vemurafenib or encorafenib in patients with BRAF-mutant melanoma (COLUMBUS): a multicentre, open-label, randomised phase 3 trial. Lancet Oncol. 2018, 19, 603-615, doi:10.1016/s1470-2045(18)30142-6.
6. European Medicines Agency Public Assessment Report. Mekinist (Trametinib) and Tafinlar (Dabrafenib) EMEA/H/C/WS/0736. Available online: https://www.ema.europa.eu/documents/variation-report/mekinist-epar-assessment-report-variation_en.pdf (accessed on 11 January 2019).
7. European Medicines Agency Public Assessment Report. Cotellic EMA/685908/2015. Availabe online: https://www.ema.europa.eu/documents/assessment-report/cotellic-epar-public-assessment-report_en.pdf (accessed on 20 December 2018).

8. Larkin, J.; Ascierto, P.A.; Dreno, B.; Atkinson, V.; Liszkay, G.; Maio, M.; Mandala, M.; Demidov, L.; Stroyakovskiy, D.; Thomas, L., et al. Combined vemurafenib and cobimetinib in BRAF-mutated melanoma. *N. Engl. J. Med.* **2014**, *371*, 1867-1876, doi:10.1056/NEJMoa1408868.
9. Novartis Pharmaceuticals Corporation. *Tafinlar [package insert]*; Novartis Pharmaceuticals Corporation: East Hanover, NJ, USA, 2018.
10. Genentech USA Inc. *Cotellic [Package Insert]*; Genentech USA Inc.: South San Francisco, CA, USA, 2018.
11. Robert, C.; Karaszewska, B.; Schachter, J.; Rutkowski, P.; Mackiewicz, A.; Stroyakovskiy, D.; Lichinitser, M.; Dummer, R.; Grange, F.; Mortier, L., et al. 3301 Two year estimate of overall survival in COMBI-v, a randomized, open-label, phase III study comparing the combination of dabrafenib (D) and trametinib (T) with vemurafenib (Vem) as first-line therapy in patients (pts) with unresectable or metastatic BRAF V600E/K mutation-positive cutaneous melanoma. *Eur. J. Cancer* **2015**, *51*, S663, doi:10.1016/S0959-8049(16)31820-2.
12. Novartis Pharmaceuticals. *Mekinist [package insert]*; Novartis Pharmaceuticals: East Hanover, NJ, USA, 2018.
13. Array BioPharma Inc. *Braftovi [package insert]*; Array BioPharma Inc.: Boulder, CO, USA, 2018.
14. Array BioPharma Inc. *Mektovi [package insert]*; Array BioPharma Inc.: Boulder, CO, USA, 2018.

© 2019 by the authors. Licensee MDPI, Basel, Switzerland. This article is an open access article distributed under the terms and conditions of the Creative Commons Attribution (CC BY) license (http://creativecommons.org/licenses/by/4.0/).
